# Synaptic Functions of Invertebrate Varicosities: What Molecular Mechanisms Lie Beneath

**DOI:** 10.1155/2012/670821

**Published:** 2012-05-13

**Authors:** Carlo Natale Giuseppe Giachello, Pier Giorgio Montarolo, Mirella Ghirardi

**Affiliations:** ^1^Department of Neuroscience, University of Torino, Corso Raffaello 30, 10125 Torino, Italy; ^2^Istituto Nazionale di Neuroscienze, Corso Raffaello 30, 10125 Torino, Italy

## Abstract

In mammalian brain, the cellular and molecular events occurring in both synapse formation and plasticity are difficult to study due to the large number of factors involved in these processes and because the contribution of each component is not well defined. Invertebrates, such as *Drosophila, Aplysia, Helix, Lymnaea,* and *Helisoma*, have proven to be useful models for studying synaptic assembly and elementary forms of learning. Simple nervous system, cellular accessibility, and genetic simplicity are some examples of the invertebrate advantages that allowed to improve our knowledge about evolutionary neuronal conserved mechanisms. In this paper, we present an overview of progresses that elucidates cellular and molecular mechanisms underlying synaptogenesis and synapse plasticity in invertebrate varicosities and their validation in vertebrates. In particular, the role of invertebrate synapsin in the formation of presynaptic terminals and the cell-to-cell interactions that induce specific structural and functional changes in their respective targets will be analyzed.

## 1. Introduction

Interneuronal communication is essential for all nervous system functions. Neurons transmit their signals to one another at specialized structures termed synapses in which a presynaptic and a postsynaptic compartment are both morphologically and functionally distinguished.

Cellular accessibility and the relative simplicity of their nervous system have made invertebrate models, such as *Aplysia, Lymnaea*, *Hirudo*, *Helix, *and *Helisoma *[1–9], a particularly suitable solution for investigating the formation of synapse and the specificity of neuronal connectivity. A large number of invertebrate neurons can be individually identified and isolated in cell culture, since they share similar size, position, biophysical properties, synaptic connections, and physiological functions among animals of the same species [10]. Therefore, it is even possible to reconstruct in dissociated cell culture synapses between individually identified invertebrate neurons that recapitulate *in vitro* their *in vivo* features [6, 7, 11–13]. Culture conditions, specific to each system, promote the regeneration of new neuritic arbors and the establishment of synaptic connections with remarkable accuracy. Thus, cell culture approaches combined with imaging and electrophysiological techniques have allowed neuroscientists to monitor cellular and molecular events underlying neurite outgrowth, synaptogenesis, and synaptic plasticity.

Commonly, vertebrate neurons display a stereotyped polarity in which it is possible to identify well-distinguished areas deputed for receiving and integrating synaptic inputs (dendrites, soma, and proximal axon), for action potential initiation (axon initial segment), and signal propagation (axonal arborization). On the other hand, invertebrate neurons normally lack myelinated axons, and their afferent and efferent processes often branch from the same offshoot of the soma. Although the presence of spine-like processes along dendrites of *Drosophila* visual interneurons [14] and honeybee calycal interneurons has been observed [15, 16], there is no evidence that neurons of other invertebrate models bear dendritic spines with a well-defined morphology as described in vertebrates. Invertebrate synapses are clustered onto varicose-like structures that appear as irregular small swellings distributed along neurites. Varicosities have been described in both invertebrate and vertebrate models, such as *Helix *[7, 17–19],* Aplysia *[20–24], *Lymnaea *[25], *Helisoma* [26], rat cortical neurons [27], pyramidal neurons [28], and hippocampal neurons [29, 30].

## 2. Initial Steps in the Formation of Varicosities

**Figure 1:**
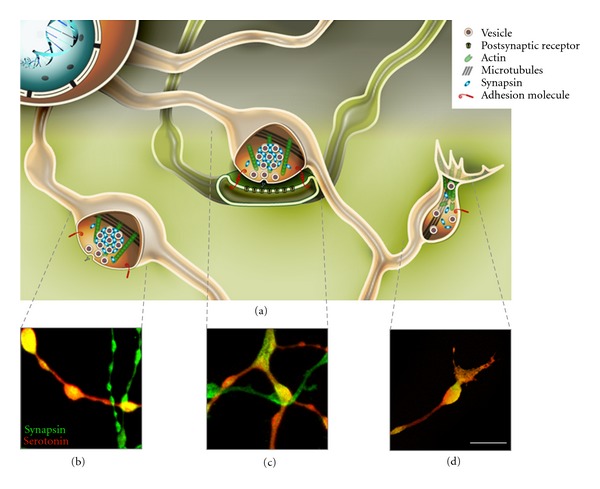
Schematic representation of the three most common types of varicosities observed in invertebrate neuronal cultures (a). The bottom panels show confocal acquisitions of neurites belonging to the serotonergic *Helix* neuron C1 cocultured with its physiological target B2 and immunostained with anti-serotonin (red) and anti-synapsin (green) antibodies. In these sample images it is possible to identify a varicosity without a postsynaptic target in which neurotransmitter release can be detected using functional dyes or electrophysiological techniques (b); a presynaptic varicosity interconnected with its postsynaptic counterpart (c); a newly formed varicosity derived from an advancing growth cone in which well-defined synaptic vesicle pools have not yet organized (d). Scale bar: 10 *μ*m.

Studies in culture have revealed that varicosities can result from the transformation of growth cone into synaptic terminal after the contact of a postsynaptic cell [31, 32], as well as along axons even in the absence of a postsynaptic target [27, 29, 33–42] (Figure 1). The formation of functional active zones lacking postsynaptic partners may be attributed to substances used for coating culture surfaces (such as polylysine, polyornithine, and basic growth factor) [37, 43–46], nevertheless this configuration is observed *in vivo* in many invertebrate [47] and mammalian central nervous system, that is, climbing fibers in cerebellum [48], mossy fibers of the dentate gyrus [49], and primary visual cortex of adult macaque [50].

In this way, the presence of multiple presynaptic regions that are dispersed along the length of the axon allows a single neuron to form *en passant* synaptic connections with many partners. Thus, we can infer that synapse formation is not a simple result of a physical contact among neurons. Interestingly, it has been demonstrated that *Helisoma *buccal neuron B5 can form an efficacious chemical synapse with B19 neuron [13, 51] within minutes after contact [3]. This mechanism does not imply a target-dependent induction of secretory capabilities. In fact, in neuron B5 the release machinery is assembled through intrinsic developmental mechanisms prior to contact [52]. Moreover, cultured *Xenopus* spinal neurons, rat hippocampal neurons, and *Drosophila* neurons show some ability for evoked synaptic vesicle recycling along entire axonal segments, even in the absence of their targets [29, 33, 35–37, 39–41]. Morphological studies performed on *Aplysia* sensory neurons cultured in contact with postsynaptic neurons as well as in isolated configuration suggest that varicosities are formed either at the tips of advancing growth cones, or along neurites after their advancement, or by splitting of pre-existing varicosities [23, 24, 53, 54].

Actually, the model proposed in the literature [55] includes a series of hierarchical steps that occur through a combination of vesicle trafficking and local recruitment of synaptic proteins. Firstly, a huge accumulation of organelles leads to vesicle cluster formation at the palm of advancing growth cone. During the assembly of presynaptic boutons, clusters of pleiomorphic vesicles have been observed at newly forming synapses [56]. Synaptic vesicle clustering to actin cytoskeleton and the following reorganization in synaptic pools may cause the sequestration of G-actin and other proteins with the consequent slowing of neuritic extension and the swelling of the central area of growth cone. Afterwards, the supply or resources are resumed, and the growth cone may carry on its advance, leaving behind a new varicosity. Finally, the newly formed varicosity is further supplemented with organelles delivered along the axons by anterograde transport. Varicosities host a heterogeneous population of subcellular organelles that include clear and dense core vesicles, mitochondria, and endoplasmic reticulum [54]. Electron microscope studies revealed that the content of varicosities formed by neurons grown in the absence of postsynaptic partners ranges from organelle high-density varicosity to those that are almost free of organelles [57, 58].

## 3. Molecular Mechanisms at Presynaptic Level: Role of Synapsin

At presynaptic level, synapsins have a prominent role in regulating the formation and the maturation of new varicosities. Synapsins are a family of synaptic vesicle-associated phosphoproteins identified in a wide range of vertebrate and invertebrate organisms [59–63]. These proteins are predominantly localized at the surface of synaptic vesicles [64–66] and maintain vesicle pool organization tethering synaptic vesicles to actin cytoskeleton. Thus, vesicle mobilization may be regulated by synapsins in a phosphorylation-dependent manner. Real-time imaging in hippocampal cultures has demonstrated that phosphorylated synapsin dissociates from vesicle clusters during tetanic stimulation [67], delivering vesicles from reserve pool to replenish the readily releasable pool, which has been depleted upon activity [68]. Synapsins are multidomain proteins sharing a common N-terminal region composed of three domains (domains A, B, and C) that are highly conserved across isoforms and species with the exception of domain B. The C-terminal domain composition (D-I) is more variable and derives from alternative splicing events [61, 69].

While in mammals the different isoforms of synapsin proteins are coded by three distinct genes, invertebrates and lower vertebrates contain only one single gene. It may be plausible that synapsin family originates from one ancestral precursor, which was subjected to duplication events when vertebrates diverged from invertebrates [61]. The hypothesis of an ancestral single synapsin gene has been validated after cloning and sequencing of synapsin in some invertebrate species such as two ecdysozoans, the fly *Drosophila melanogaster *[70] and the nematode *Caenorhabditis elegans* [61], and three lophotrochozoans, the mollusks *Loligo pealei* [71], *Aplysia californica* [72], and *Helix pomatia* [73]. Therefore, the evolution of these proteins in the different phyla correlates with the development of a progressively more complex nervous system.

There are many pieces of evidence that synapsins play a role in axon elongation and synapse formation. It has been demonstrated that synapsin I and II regulate synaptic functions following the early neurogenesis in mouse brain [74]. Synapsin III is expressed mainly in early phases of neuronal development and is highly concentrated in growth cones [75]. Moreover, the onset of presynaptic maturation at *Xenopus* neuromuscular junctions is causally related to the onset of synapsin expression [76], indeed experimentally elevated levels of synapsin I [77] or synapsin IIa [78] accelerate presynaptic maturation characterized by a precocious assembly of active zone structures, organization of synaptic vesicle pools, and also a rapid formation of thickenings of postsynaptic muscle cell membrane [79]. Interestingly, even the overexpression of synapsin in non-neuronal cells results in the formation of neurite- and synaptic-like structures similar to varicosities [80].

As in vertebrates, synapsin expression correlates well with the time course of presynaptic terminal maturation and synaptogenesis in mollusks, such as *Helix *and *Aplysia. *In isolated *Helix* neurons, immunostaining for mammalian synapsin I appears uniformly distributed in the cell body, the distal axonal segments and the growth cones. The contact and the formation of a chemical connection with an appropriate juxtaposed target induce a redistribution of the synapsin mainly in varicosity-like structures immunoreactive for the neurotransmitter serotonin along neurites close to the target neuron [19], similar to the changes in synapsin I distribution following synaptic contacts in hippocampal neurons developing in culture [81]. Interestingly, the number of synapsin-positive varicosity-like structures increases progressively parallel to the enhancement in the mean amplitude of the postsynaptic potentials recorded at the same times in *Helix* cocultures.

A distribution of synapsin in distinct puncta along neurites has also been shown in neurons of *Aplysia punctata* [19] and of *Aplysia californica* [72] where synapsin undergoes dispersion following serotonin and TGF*β*1 treatments that induce its phosphorylation mediated by PKA [72] and MAPK kinases [82], respectively. Therefore, the phosphorylation of *Aplysia* synapsin may result in its dissociation from synaptic vesicles in processes activated by neuromodulators and growth factors involved both in modulation of neurotransmitter release and in remodelling of growing neurons during development.

To study the synaptic fidelity of neuronal regenerating connections in culture, a multitude of experiments about target recognition during synaptogenesis has been performed in invertebrate neurons [4, 83–87]. Identified motoneurons, isolated from the buccal ganglia of *Helisoma trivolvis*, display selective synapse formation in culture [3, 51]. In particular, the identified B19 neuron forms appropriate cholinergic connections with buccal muscle fibers, but not with other buccal neurons [88].

In *Helix* nervous system the giant metacerebral neuron C1, homologous of the *Aplysia *MGC [89], physiologically forms a serotonergic monosynaptic connection with the giant neuron B2 in the buccal ganglia [90]. *In vitro* studies demonstrated that the presence of a non-physiological target neuron C3 results in a general inhibitory effect on the maturation of the presynaptic terminals of neuron C1, reducing both the number of presynaptic varicosities and their ability to release neurotransmitter in the presynaptic neuron [91], through mechanisms that involve the downregulation of both MAPK/Erk and PKA pathways [92]. These pathways are rapidly activated by the contact with the physiological target neuron B2 that can quickly reverse the wrong target-induced inhibition.

In *Helix *C1 neurons cultured in contact with a wrong target C3, injection of bovine synapsin I has been shown to exert an enhancing effect on the efficiency of the neurotransmitter release machinery [93]. Interestingly, the injected synapsin was able to rescue neurotransmitter release strongly depressed by the presence of the non-physiological target bringing it to levels comparable to those observed when the C1 neuron is cocultured with its physiological target B2 [91]. This suggests that exogenous synapsin I may accelerate the maturation or simply disinhibit the quantal release mechanisms by affecting cytoskeleton assembly and/or synaptic vesicle clustering in *Helix *presynaptic terminals, in agreement with the results obtained by injection of synapsin I or II into embryonic *Xenopus *neurons that accelerates both the morphological and functional development of synapses [77–79].

## 4. Synapsin Phosphorylation and Synapse Formation

Further experiments in *Helix* neurons in culture have analyzed the role of specific domains of synapsin in regulating structure and activity of synapses [73, 94, 95]. A multiple alignment of the primary structure of *Helix* synapsin [73] with *Aplysia* synapsin [72] and other mammalian orthologs reveals the high phylogenetic conservation of the PKA/CaMKI/IV phosphorylation site located in the N-terminal domain A (Ser-9). The phosphorylation of this site by either PKA or CaMKI/IV is necessary for the enhancement of neurotransmitter release from neuron C1 to overcome the inhibitory effect of the wrong target in C1–C3 soma-soma coculture, since the facilitating effect due to the injection of exogenous wild-type synapsin is maintained in the presence of the pseudo-phosphorylated form and virtually lost after the injection of the non-phosphorylatable mutant. Moreover, the functional effects of gastropod synapsin are associated with a phosphorylation-dependent ultrastructural rearrangement of neurons C1. In fact, electron microscopy analysis showed that in the region of contact between C1, overexpressing wild-type synapsin, and neuron C3 there were dense interdigitations of microtubule-packed neurite-like processes with the appearance of dense core synaptic vesicle clusters typical of C1, that were virtually absent in uninjected C1–C3 pairs or after injection of the nonphosphorylatable domain A mutant [94].

Studies on vesicle dynamics in growth cones [96] show a critical role of the PKA phosphorylation site in synapsin I, suggesting that the same molecular mechanisms involved in modulating neurotransmitter release from mature nerve terminals may also underlie the activity of the protein in developing terminals. The increase in cAMP in the presynaptic terminal following the contact with the postsynaptic target [97] may regulate synapsins activity in the control of synaptic vesicle distribution and recycling leading to the transformation of the growth cone into a mature presynaptic bouton. Recently, the overexpression of synapsin domain A phosphomutants in mice lacking endogenous synapsins has restated that this phosphorylation site plays an important role in controlling synapses formation. While the presence of the pseudo-phosphorylated form can accelerate synapse formation, the overexpression of the non-phosphorylatable mutant may cause a significant decrease in the total amount of both glutamatergic and GABAergic synapses during development [98].

In addition to PKA pathway, the phosphorylation of synapsin by MAPK/Erk kinase has also a critical role in the formation of synapses between *Helix* neurons in culture [95], consistent with a lot of evidence supporting the role of MAPK/Erk kinase in neurotrophic regulation of synapse formation and plasticity in vertebrate and invertebrate models [99–104]. *Helix* synapsin bears two putative MAPK/Erk consensus sites in domain B, Ser36 and Ser42, that are highly conserved among the known invertebrate synapsin proteins and might represent homologous MAPK/Erk phosphorylation sites to sites 4 and 5 of mammalian synapsin [105].

Overexpression of both MAPK/Erk phosphomutants induce a significant reduction of the presynaptic differentiation of the injected neuron and of the number of synaptic connections between the paired cells. In addition, the basal amplitude of the postsynaptic potentials recorded in *Helix* B2-B2 neurons is markedly reduced following injection of the non-phosphorylatable MAPK/Erk mutant while it is slightly decreased by the injection of the pseudo-phosphorylated MAPK/Erk mutant. Both mutants have no effect on the rising time of the postsynaptic potentials and do not induce any changes in the neurite outgrowth, suggesting that the reduction in synaptic strength occurs in the absence of changes in neurotransmitter release kinetics and ruling out the possibility that the altered connectivity depends on impairment in neurite growth. These observations suggest that MAPK-dependent synapsin phosphorylation regulates the occurrence of chemical synapses through a growth-independent mechanism [95]. The similar negative effect of both non-phosphorylatable and pseudo-phosphorylated synapsin mutants on synaptic formation suggests that cycles of MAPK/Erk phosphorylation may play a fundamental role in regulating synapsin activity during synaptogenesis, perhaps acting on cytoskeletal assembly and vesicle clustering at synaptic terminals, as suggested by the role of MAPK/Erk phosphorylation in modulating synapsin affinity for actin [105].

All the effects observed with mutagenesis experiments described so far cannot be ascribed to mistargeting of synapsin localization. Confocal acquisitions of soma-soma *Helix* neurons cocultures overexpressing GFP-conjugated synapsin phosphomutants for either PKA/CaMKI/IV or MAPK/Erk in the presynaptic compartment display a deeper clusterization pattern but the same localization observed for the wild-type form. In particular, ectopic synapsins are present in presynaptic neurons with a preferential localization in the contact area with the postsynaptic target and along presynaptic neurites projecting onto the postsynaptic cell, in the areas of the soma-soma pairs containing the majority of the synaptic vesicle clusters and synaptic structures. Therefore, the phosphorylation of the synapsin N-terminus seems not to be implicated in the correct targeting of *Helix* synapsin, consistent with the observation in cultured hippocampal neurons where the deletion of synapsin domain A does not significantly impair the synaptic targeting of mammalian synapsins [106]. The higher degrees of clustering of overexpressed GFP-tagged synapsin mutants compared with the wild-type protein are possibly due to a stronger association of synapsin mutants with synaptic vesicles and/or to a lower rate of its dispersion and reclustering cycles [67, 107]. Conversely, the pseudo-phosphorylated mutants show a very low degree of clustering and appear uniformly diffuse along neurites. These observations are consistent with previous morphological studies showing that serotonin-induced dispersion of synapsin clusters in *Aplysia* neurons depends on both PKA and MAPK/Erk activity [72], and that PKA and MAPK/Erk phosphorylation regulates the mobility of synapsin as well as the trafficking of synaptic vesicles in nerve terminals upon stimulation [108].

## 5. Synapsin Phosphorylation and Plasticity

For many years the studies of synapsin functions have been focalized on synaptic plasticity rather than synaptogenesis. Synapsin proteins are implicated in maintenance of presynaptic vesicular pools and in the regulation of vesicle mobility among them during short-term plasticity [109–111]. In particular, synapsins appear to have a fundamental role in the expression of post-tetanic potentiation (PTP) since both genetically altered mice and *Aplysia* synapses exhibit a marked impairment of PTP after genetic deletion or neutralization of synapsin I and/or synapsin II [109, 112]. Interestingly, synaptic vesicle mobilization from the reserve pool during PTP in *Drosophila* is strongly dependent on PKA activation [113, 114]. Phosphorylation of synapsin domain A might also modulate PTP by altering the presynaptic release probability as shown in PTP at the calyx of Held synapse [115] that may be mediated by the activation of CaMKs [116]. However, microinjection of domain A peptide into the squid giant synapse had no effect on vesicle pool size, synaptic depression, or transmitter release kinetics, indicating that this domain may be predominantly involved in regulating synaptic vesicle trafficking at pre-docking stages [117]. Presynaptic overexpression of the *Helix* synapsin non-phosphorylatable mutant in domain A specifically impairs PTP, while the overexpression of the wild-type form has no effect on peak amplitude or time course of PTP [73]. Similarly, these results have been confirmed at *Aplysia* synapses [118]. In addition, PTP expression in *Helix* neurons critically depends on MAPK/Erk activation [95], which might occur upon intracellular calcium build-up during the tetanus [119, 120] or via crosstalk with other calcium-dependent pathways [121–124]. Although MAPK/Erk activity does not appear to be required for short-term heterosynaptic facilitation induced by serotonin at *Aplysia* sensory-motor synapses [8, 125, 126], other studies in invertebrates show that modulation of short- and long-term synaptic plasticity paradigms is mediated by MAPK/Erk [82, 127–129]. An involvement of MAPK/Erk in short-term plasticity is also supported by studies in transgenic mice that express a constitutively active form of H-Ras, which exhibit an enhancement of paired-pulse facilitation and long-term potentiation that is dependent on MAPK/Erk activation [130].

As demonstrated by the studies described above, the same molecular pathways and effectors that regulate the formation of functional synaptic contacts are also involved in synaptic transmission and plasticity. Even if these mechanisms acting at presynaptic level seem to be intrinsically regulated, the presence of a target cell during synaptogenesis has a prominent role in triggering the formation and maturation of specialized structures in both pre- and postsynaptic neurons.

## 6. Synapse Formation: Crosstalk between Pre- and Postsynaptic Sites

At postsynaptic level, synapse formation requires a coordinated assembly of synaptic structures conferring the competence to translate the presynaptic signal into a postsynaptic response. In vertebrate neurons, two important steps are the formation of a protrusion that differentiates into a dendritic spine and the formation of a postsynaptic density facing the active zone. These events require the involvement of a multitude of different proteins, which have been partially identified and characterized. Actually, several models have been proposed in spinogenesis: spines may derive either from the stabilization of an initial filopodium after the contact with the axon [131–133], or from filopodium-independent sprouting [134, 135], or, alternatively, they might initially grow without synaptic contact [136–142].

Considering the requirement of a synaptic contact, several protein families have been proposed to trigger spinogenesis mediating cell-to-cell communication, such as cadherins, neuroligin-*β*-neurexin cell adhesion complexes, and ephrins/Eph receptors [143–145]. Although these molecules have been shown to play a role in the various aspects of synaptogenesis, matching pre- and postsynaptic components, no single protein factor has been found to be essential for all these processes, from initial synapse specification to the formation of functional connections.

Cadherins are a large family of Ca^2+^-dependent, homophilic, cell-surface adhesion molecules [146–152]. Both E-cadherins and N-cadherins are present in synapses, and they are symmetrically localized in the adhesive junctions that surround the active zone in the presynaptic terminal and the postsynaptic density [146]. In cultured hippocampal neurons, N-cadherin is ubiquitary expressed in all synapses only at early stages of development, then becomes restricted to a subpopulation of excitatory synapses during maturation [153]. Recent studies have linked these proteins to dendritic spine morphogenesis. A delay in spine formation has been observed in cultured hippocampal neurons overexpressing a dominant-negative form of N-cadherin, lacking part of the extracellular domain. Although the loss of N-cadherin activity promotes the appearance of immature filopodia with irregular shapes, synaptic contacts are retained. Moreover, the presence of dominant-negative N-cadherin impairs the localization of both presynaptic and postsynaptic protein markers, that is, synapsin and PSD-95, respectively [154]. This effect seems to be more pronounced at early stages of synaptogenesis, suggesting that cadherins may be more involved in synapse formation rather than stabilization and maturation.

Nevertheless the role of classical cadherins in triggering synapse formation is still debated. Indeed genetic studies in *Drosophila* has greatly contributed to determining the function of N-cadherins *in vivo*. Loss of N-cadherin in *Drosophila* embryos affects the trajectories of longitudinal CNS axons and the guidance of growth cones [155]. It has been demonstrated that N-cadherin is important for coordinating the targeting of multiple neuronal types, such as R7 photoreceptor axons and L1–L5 lamina neurons, to the right target layer in the medulla neuropil of the visual system [156–159]. *Drosophila* contains 12 isoforms of N-cadherin, but the expression of a single isoform is sufficient to rescue null mutations, suggesting functional redundancy [159]. Thus, these observations indicate that cadherins may be involved in target recognition and perhaps stabilization of early synaptic contact sites but not in the induction of synapse formation.

Another protein, neuronal-cell adhesion molecule (N-CAM), belonging to the Ca^2+^-independent cell adhesion molecules of the immunoglobulin superfamily, is also present in synapses [160–164]. This protein bears fibronectin type III repeats in the extracellular domain and a short cytoplasmic domain, anchored to the cytoskeleton, which interact with intracellular signaling pathways [165, 166]. *In vitro* studies showed that several identified CAM members regulate the number of synaptic contacts, their morphology and functions; however a strong evidence that any of these molecules is necessary for synapse formation *in vivo *is lacking, probably suggesting a redundancy in their functions. In cell cultures, N-CAMs accumulate quickly at sites of contact formation during the initial assembly of synaptic components [167]. Through interaction with spectrin-coated trans-Golgi-derived organelles, N-CAM may promote the accumulation of those postsynaptic proteins that are necessary to form the synaptic contact [168]. In fact, a reduction in size of postsynaptic densities and an impaired recruitment of spectrin, NMDA receptors, and CaMKIIa to the synapse is observable in neurons lacking N-CAM [169]. Furthermore, studies on mixed cultures of hippocampal neurons from N-CAM knockout and wild-type mice have revealed that postsynaptic N-CAM promotes the formation and increases the strength of excitatory synapses in concert with NMDA receptor activity [170].

In literature a large amount of evidence that suggest the involvement of N-CAM not only in neuronal development, but also in synapse plasticity, results from invertebrate models [171–179]. In *Drosophila, *the concentration of fasciclin II, homologue of vertebrate N-CAM, regulates sprouting and the capability of neurons to form new synaptic contacts [177, 178]. In nerve-muscle cocultures from *Xenopus *embryos, the percentage of functional neuromuscular contacts is decreased by means of antibody against N-CAM [180].

In *Aplysia*, apCAM is predominantly expressed at synaptic contacts [172, 181] and modulates synapse formation and long-term plasticity at sensory-motor synapses [172, 179, 182–186]. The ability of sensory neuron to form *in vitro* chemical connections with motoneuron L7, detected as number of branches and varicosities, correlates with the expression level of apCAM on different regions of the postsynaptic cell L7 [184]. Moreover, a reduction in fasciculation of growth cones has been observed with the preincubation of isolated sensory or motoneurons with a monoclonal antibody against apCAM [172, 181, 187]. While the addition of the antibody on preformed sensory-motor cocultures results in a failure of serotonin-induced long-term changes in synaptic efficacy and the concomitant morphological changes of sensory neuron, such as formation of new varicosities, without altering the transmission of pre-existing synapses and their short-term modulation [185]. Interestingly, the same anti-apCAM antibody recognizes apCAM-like proteins of the *Helix* nervous system. The neurotransmitter releasing ability of *Helix* neuron C1 is detectable when it is cultured alone or in presence of its physiological target B2, whereas it is inhibited by the presence of the wrong target C3 [19, 91]. In C1–C3 cocultures, the buildup of neurotransmitter release triggered by the appropriate target B2 is prevented by preincubation of this neuron with anti-apCAM antibody [188], confirming that N-CAM orthologs may play an important role during the contact of two synaptic partners in modulate the efficiency of excitation-secretion coupling.

One potential signaling cascade implicated in this phenomenon is PKC [189, 190], since the presence of apCAM on membrane of motoneuron L7 and the activation of *Aplysia* PKC isoforms PKC *Ap*l II are both necessary events for the initial synapse formation and the increase of sensorin expression by sensory neurons [191]. Therefore, apCAM exposed on L7 membrane surface may activate signaling cascades not only in the motor neuron itself, but also in the coupled sensory neuron via the heterophilic receptor to regulate both pre- and postsynaptically the expression of effectors necessary for the assembly of functional synapses [169, 192–197].

Based on invertebrate *Drosophila* Fasciclin II and *Aplysia* apCAM sequences, a database-search analysis resulted in an identification of a similar protein in vertebrates, called SynCAM [198]. SynCAM is a transmembrane member of the Ig superfamily that mediates Ca^2+^-independent homophilic interactions and displays a structure similar to the nectins [199]: 3 Ig-domains followed by an intercellular C-terminal PDZ-binding motif able to bind the synaptic scaffolding proteins CASK and syntenin. High level of SynCAM expression has been detected in young rat brain in the first few weeks after birth, corresponding with the main period of synaptogenesis. Overexpression studies in cultured hippocampal neurons confirmed that SynCAM promotes synapse formation and increases spontaneous synaptic activity while its isolated cytoplasmic tail inhibits synaptic function, perhaps by acting as a dominant negative [198]. Remarkably, this protein has the ability to promote the formation of active presynaptic terminals in non-neuronal cells, when cocultured with hippocampal neurons [198]. Hence, SynCAM may act at multiple stages of synaptogenesis from the initial synaptic contact to the modulation of neurotransmitter release. However, its effects on dendritic spine morphology remain to be determined.

## 7. Synapse Modulation and Plasticity: Role of Adhesion Molecules

Once a functional contact is established, the new synapse goes through a series of maturation processes that is likely to be regulated by neural activity. For example, hippocampal synapses undergo structural changes after long-term potentiation (LTP) *in vitro* and experience *in vivo* [200, 201]. Generally, at postsynaptic level, newly formed spines acquire a postsynaptic density and increase their volume which closely correlates with the exposure in membrane of additional AMPA receptors [202] and the reorganization of the actin cytoskeleton [203]. These processes are strictly associated with the induction of LTP [139, 200, 204–206].

Before the large number of data collected from hippocampal neurons about the involvement of adhesion molecules, such as N-CAM, in long-term potentiation [160, 161, 166, 176, 207–211], early studies about long-term modifications were performed on invertebrate models. In particular, an important step in our understanding of N-CAM functions comes from studies on long-term functional and structural plasticity of the *Aplysia* sensory-motor synapse. ApCAM is expressed at the highest levels at sites of synaptic contact between sensory and motor neurons in culture, consistent with its *in vivo* distribution [181]. Long-term facilitation induced by serotonin application is accompanied by the formation of new branches and varicosities in sensory neuron [21, 212]. On the other hand, long-term depression of the same synapse by the neuropeptide Phe-Met-Arg-Phe-amide (FMRFamide) is correlated with the loss of presynaptic sensory neurites and varicosities [213, 214]. Both modifications of synaptic efficiency involve a rapid and cell-specific change in the distribution of apCAM. The treatment with 5-HT causes a downregulation of apCAM from the surface of the sensory neuron via a cAMP-dependent increase in endocytosis of clathrin-coated vesicles [172, 182, 215], while application of FMRFamide induces a downregulation of apCAM from the surface of the target motor neuron by a similar cAMP-dependent mechanism [183, 187]. Consistent with these observations, transgenic mice in which N-CAM has been depleted showed deficits in learning and memory [216]. Furthermore, the interference with N-CAM levels through specific antibodies or suppression of NCAM results in a reduced or even abolished LTP in the CA1 region of the hippocampus [161, 176, 217].

## 8. Concluding Remarks

Synaptogenesis is a complex process that results in the assembly of a functional release machinery in the presynaptic terminals and the formation of specialized structures at the corresponding postsynaptic level. In recent years, considerable progress has been reached in understanding the cellular and molecular mechanisms of vertebrate synaptogenesis. New techniques and approaches have allowed scientists to characterize several molecules that regulate not only when and where synapses are formed but also their continuous plastic modifications. Beside this, it is important to mention the contribution of pioneering experimental studies performed on invertebrate models that permitted the identification of the basic mechanisms of neuronal functions implicated in behavioral responses that are phylogenetically conserved in vertebrate animals.
